# Sero-prevalence for Hepatitis B virus among pregnant women attending antenatal clinic in Juba Teaching Hospital, Republic of South Sudan

**DOI:** 10.11604/pamj.2017.26.72.11410

**Published:** 2017-02-20

**Authors:** Anthony Laku Stephen Kirbak, Zipporah Ng’ang’a, Jared Omolo, Hakim Idris, Abdulmumini Usman, William Baguma Mbabazi

**Affiliations:** 1 EPI and Child Health Department, Ministry of Health, P.O Box 88, Juba, Republic of South Sudan; 2 Jommo Kenyatta University of Agriculture and Technology, P.O Box 62000-00200 Nairobi, Kenya; 3 Juba Teaching Hospital Laboratory, Ministry of Health, P.O Box 88, Juba, Republic of South Sudan; 4 World Health Organization, P.O Box 82, Juba, Republic of South Sudan; 5 CDC/AFENET Immunization workforce development project, P.O Box 88, Juba, Republic of South Sudan

**Keywords:** Hepatitis B prevalence, pregnant women, South Sudan

## Abstract

**Introduction:**

Hepatitis B virus infection is a major public health problem worldwide and in Africa. This would be the first ever documented study on epidemiology of Hepatitis B infections in the newly formed Republic of South Sudan. This study was designed to estimate the sero-prevalence of Hepatitis B virus infection amongst pregnant women attending antenatal services in Juba.

**Methods:**

A cross-sectional study was conducted among pregnant women attending antenatal clinic services in Juba Teaching Hospital, in the period between December 2012 and March 2013. Any pregnant woman, attending antenatal care services at Juba Teaching Hospital, was included if she was a resident of Juba County for at least 1 year before pregnancy. A Hepatitis B case was defined as any women participating in the study and was found to be positive for HbsAg and confirmed by ELISA.

**Results:**

This study documented that the prevalence of Hepatitis B surface antigen (HBsAg) among pregnant women attending ANC in Juba was 11% (31 out of the 280 samples). Other samples tested were indeterminate (36%), naturally immune (27.1%), susceptible (23%) and the remaining 1.8% was immune due to vaccination. Significant risk factors for Hepatitis B infection were loss of partner (OR 4.4 and CI of 1.4-13.9) and history of Jaundice (OR 1.7 and CI of 1.2-2.1).

**Conclusion:**

These study findings show that only 29% of infants in Juba county are born to immune mothers (naturally or vaccine induced). The remaining 70% of babies would be at risk of infection, if a birth dose of Hepatitis B is not provided. We therefore recommended introduction of Hepatitis B Vaccine birth dose into routine infants’ vaccination series to eliminate this risk.

## Introduction

Chronic Hepatitis B virus (HBV) infection affects 400 million people worldwide and is the most common cause of liver cirrhosis and hepatocellular carcinoma [1]. Vertical transmission of HBV is the main cause of chronic HBV infection and is a problem in endemic areas, such as the Far East and Africa [2–8]. In 1992, the Global Advisory Group to the World Health Organization (WHO) recommended that all countries integrate hepatitis B vaccine into national immunization programs by 1997. As a result, several countries introduced hepatitis B vaccination in their routine immunization program in combination with DTP vaccines and may have increased the opportunity to prevent peri-natal transmission of HBV. The Strategic Advisory Group of Experts (SAGE) on immunization meeting in October documented that 185 (95%) countries worldwide had introduced hepatitis B vaccination in their infant schedules with only 97 (49%) countries providing the recommended birth dose [9].

Like other countries, South Sudan introduced hepatitis B vaccination into the National infant Immunization program using the Pentavalent (DTP-HepB-Hib) vaccine formulation. While this policy option was considered operationally more feasible, it negates the plight of several children born with high risk of vertical transmission. Although evidence from South Sudan is scanty, HBV sero-markers studies in Khartoum using ELISA (and not DNA techniques) suggests that 5.6% of pregnant Sudanese mothers were positive for HBVsAg irrespective of their age, parity and socio-demographic characteristics [10]

In Southern Sudan the prevalence of HBVsAg was previously estimated to be 26% [10]. When mother to child transmission of HBV infection was studied in Juba, South Sudan, five out of nine (55.5%) babies born to HBsAg positive mothers were infected, documenting the high-risk of infection in early childhood [11]. Even in countries with developed health care systems, screening women for high-risk groups failed to identify 35%-65% of HBsAg positive pregnant women [12, 13] as a result, it is recommended that all pregnant women should be screened for HBsAg, [14] a practice that may not be feasible in developing health systems context.

From the public health perspective, preventing HBV infections acquired at birth and in early childhood is critical, as children have a 90% chance of becoming chronic carriers if infected at the time of birth, a 30% chance of becoming chronic carriers if infected between one and five years of age, and a 5% to 10% chance of becoming chronic carriers if infected after five years of age [15]. HBV transmission can be prevented by vaccination of at risk-infants. In the absence of a health care system that can effectively screen for HBsAg infections in pregnant mothers, it would make public health sense (Population approach) to introduce hepatitis B vaccination into childhood immunization programs that includes administration of a birth dose. In previous studies, failure of neonatal vaccination of at-risk infants (breakthrough HBV infections) was found to be associated with high viraemia of the mother [16, 17] and no evidence was found in Southern Sudan that estimates neither the levels of viraemia in pregnant mothers nor the risk of breakthrough infections. Apart from the social and political interpretations of HBV vaccination at birth, these are the two technical questions that ought to be studied prior to advancements on the policy option of HBV vaccination at birth.

South Sudan has been in war for more than 20 years, has many displaced population, internally and externally to neighboring countries including Uganda and Kenya, and still has poor primary health care structures. Lack of hepatitis B birth-dose vaccination policy in the routine infant vaccination series, and no mass campaigns conducted to at risk children, compels the country to study the prevalence of HBV infection in pregnant women. This will provide information to the authorities that will guide decisions on the appropriate vaccination strategies and age groups to be targeted (Health Sector Investment Plan, 2011-2015). The purpose of this study was to establish the sero-prevalence and risk factors of hepatitis B virus infection among pregnant women, attending antenatal services in Juba teaching hospital, South Sudan.

## Methods

### Study design and period

This was a cross- sectional study that quantitatively estimated the sero- prevalence of Hepatitis B virus in pregnant women attending antenatal clinic at Juba Teaching Hospital from December 2012 to March 2013.

### Study area, sample size determination and sampling method

The study site was Juba Teaching Hospital, in Juba County, Central Equatoria State in the Republic of South Sudan. Juba County is composed of three (3) urban payams (Juba, Kator and Munuki) and 13 other rural payams. The study population was pregnant women who were residents in Juba County for not less than 12 months, attending and using the antenatal services in Juba Teaching Hospital, during the study period.

The sample size was calculated using single population proportion formula [18] by assuming lowest prevalence of HBV 5.6% [19] and highest prevalence of 24% [19]. Accordingly, a total of 280 study participants were enrolled. Probability sampling using the systematic method was used to recruit study participants. The sampling frame consisted of all pregnant women attending ANC at juba teaching hospital. The sampling interval (K unit) was calculated as 9 mothers using ANC records that showed a monthly attendance of 860 first visit attendance, with 40% of attendees being residents of the three Urban Payams of Juba County. A random starting point was then selected. The random starting point was any number < 10 and for this study the 3rd registered woman was considered as the starting study participant. The subsequent study participant was determined by cumulatively adding the K unit.

### Data collection

Data was collected using a validated and pre-tested structured questionnaire. Interviews were conducted by the principal investigator in presence of a trained assistant female midwife, to collect socio-demographic data and other variables associated with the risk of HBV infection, after obtaining written consent from an informed client. Three-five milliliters (3-5 Mls) of whole blood was also collected from each study participant by venous puncture, and serum was separated. The serum was used for rapid serological HBsAg test and enzyme-linked immunosorbent assay (ELISA).

### Laboratory detection of hepatitis B virus

Hepatitis B surface antigen (HBsAg) and antibody (Anti-HBs, Anti-HBc IgG and Anti-HBc IgM) assays were done using the Abbot Murex enzyme immune-assay kits. Quality control references provided in the test kits were used to standardize the laboratory testing. An antibody level greater than or equal to 10 MIU per ml was considered protective against HBV infection and indicated acquired immunity [20]. For HbsAg, samples with an optical density equal to or greater than the cut-off value provided in the kit was considered reactive in the assay. Interpretation of laboratory results was based on the combination assay results as adapted from CDC interpretation of hepatitis B panel (Table 1).

**Table 1:** CDC Panel for interpretation of Hepatitis B serology results

HBsAg	Anti-HBs IgG	Anti-HBc IgG	Anti-HBc IgM	Interpretation
Negative	Negative	Negative	Negative	Susceptible
Negative	Positive	Negative	Negative	Immune due to hepatitis B vaccination
Negative	Positive	Positive	Negative	Immune due to natural infection
Positive	Negative	Positive	Positive	Acutely infected
Positive	Negative	Positive	Negative	Chronically infected
Positive	Positive	Positive	Negative	Chronic carrier
Positive	Positive	Negative	Negative	Chronic carrier with possible false negative anti HBc test
Positive	Negative	Negative	Negative	Chronic carrier with possible false negative anti HBc test
Negative	Negative	Positive	Negative	Indeterminate: *Four interpretations possible:* Maybe recovering from an acute HBV infection Distantly immune and test not sensitive enough to detect very low levels of anti-HBs in serum Carrier with undetectable level of HbsAg Susceptible with false positive anti-HBc

Source: CDC- Viral Hepatitis B. Interpretation of the hepatitis B panel.

### Quality control

The standard operational procedures (SOP) of pre-analytical to post analytical quality control techniques of the laboratory testing procedures were strictly followed. The quality control for HBsAg test kits was assured by using positive and negative control samples before testing the patients’ serum, and in every opening of new kits. Similarly, ELISA test results were also assured using positive and negative controls according to the manufacturer’s manual. The data quality was also maintained by training all midwives involved in the data collection. Lastly, daily checking (for completeness, accuracy and consistency) and quality assurance of results was done by the principal investigator.

### Statistical analysis

An epidemiological data capture questionnaire and a laboratory investigation form was developed and used during the study. All filled epidemiological and laboratory investigation forms were reviewed by the principle investigator for completeness, accuracy and consistency. The forms cleared by the principle investigator were then double-entered into EPI INFO 2003. Laboratory and questionnaire data for each participant were linked during entry. Data cleaning was conducted by the principle investigator before analysis was conducted. The major data analysis outputs were: frequency tables for study participants’ descriptions, HbsAg, antibodies against surface and core antigens. Bi-variate analysis of sero-infected pregnant women was conducted to determine risks of being infective and risk factors for Hepatitis B infection.

### Ethical considerations

This research project was approved and ethically cleared by the Ethics Review committee of the Ministry of Health, Republic of South Sudan. Written consent was also obtained from each study participant. Specimens were delinked from the participants’ identity to ensure confidentiality and blinding of the laboratory staff conducting the laboratory analysis of samples. HBsAg screening test results were communicated to each study participant before they were referred to obtain monovalent Hepatitis B vaccination. All babies born to all mother participating in the study were all provided a birth-dose of monovalent HBV vaccination.

## Results

### Study participants

**Figure 1:**
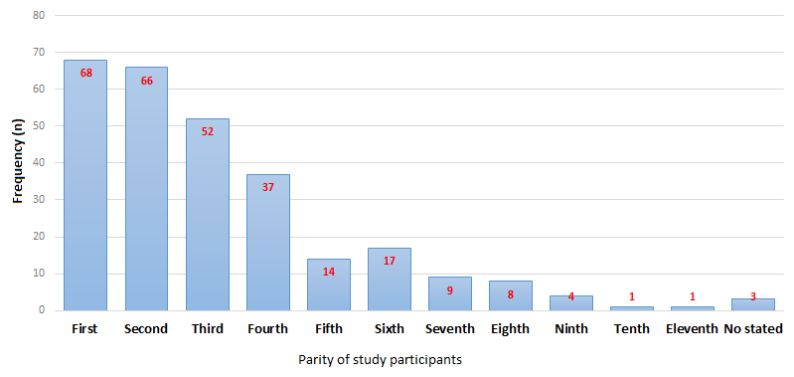
Frequency distribution graph showing parity of study participants

The study enrolled 280 pregnant women attending antenatal clinic in Juba Teaching Hospital. Their age ranged between 15 and 44 years. The youngest participant was 15 years and the oldest was 44 years. Age group 20-24 years had the highest number of participants (n=95, 33.9%), followed by 25-29 years (n= 82, 29.3%). The older group 40-44 years was the least (n= 3, 1.1%) The majority of study participants were 1st to 4th pregnancy. The modal pregnancy was prime gravidae. The least number of participants was observed in the category of 7 or more pregnancies (Figure 1). The majority (69.3%) of study participants had primary or no education at all. Nearly a quarter of the study participants had no education at all. The participants with the post-secondary education were the minority (6.4%). The majority of the study participants (65%) did not have history of surgical intervention while only 32.5% had surgical history. When asked about history of blood transfusion, a majority of study participants (93.2%) had not and only 4.6% had history of blood transfusion. Majority of the study participants (73%) were married women, 12.9% were separated/divorced, 1.8% were single and 7% did not respond to this question. Other risk factors evaluated in this study are outlined in the frequency distribution Table 2.

**Table 2:** Frequency table for Hepatitis B risk factors among study participants

Risk Factor	Frequency (n)	Percentage	Non-response (%)
Urban Residence (n=280)	181	64.6	10.0
History of scarification/tribal marks(n=280)	61	21.8	5.0
History of contact with HBV patients (n=280)	23	8.2	14.7
History of sharing sharp instruments (n=280)	35	12.5	1.1
History of Abortion (n=280)	49	17.5	15.4
History of Jaundice (n=280)	30	10.7	16.1
More than one sexual partner since debut	52	18.6	18.2
Husband has more than one wife (n=280)	66	32.4	19.1

### HBV sero-prevalence

Of the 280 study participants, whose serum samples were collected and screened only 31 (11%) were positive, with detectable levels of Hepatitis B surface antigen (Table 3). Only 270 out of the total 280 (samples were tested using confirmatory ELISA. Majority of study participants (36%) were indeterminate, naturally immune (28%) and susceptible (23%). Only 2% were immune (due to vaccination). Ten samples were not confirmed by ELISA test (Table 4).

**Table 3:** Number of serum specimen screened for HBsAg

	Frequency	Percent
HBsAg Positive	31	11.1
HBsAg Negative	249	88.9
Total	280	100

**Table 4:** Results summary of Hepatitis B ELISA assays in the study population

S/No.	Interpretation	Total	%
1	Indeterminate	98	35.0
2	Immune (naturally)	76	27.1
3	Susceptible	61	21.8
4	Infected (Acute/Chronic)	30	10.8
5	Immune (vaccinated)	5	1.8
6	Not tested for ELISA confirmation	10	3.6
	Total	280	100

### Associated risk factors

The study results were cross tabulated for bivariate analysis and showed that the risk of Hepatitis B infection were significantly associated with a) loss of marital partner (odds ratio of 4 in a 95% confidence interval of 1.39 to 13.92) and b) history of jaundice (odds ratio of 1.69 in a 95% confidence interval of 1.21-2.14). Other risk factors studied were not significantly associated with positive Hepatitis B infection rates (Table 5).

**Table 5:** Results summary of bivariate analysis of risk factors for Hepatitis B

		Positive	Negative	OR	95% CI	P-Value
*Education* (n=270)	Primary or less	21	166	1.97	0.87- 4.25	0.103
	More than primary	5	78			
*Current Occupation* (n=270)	House work	24	194	1.16	0.39-2.59	0.992
	Work outside home	5	47			
*Loss of Partner* (n=260)	Lost partner	5	10	4.40	1.39-13.92	0.006
	Not lost partner	25	220			
*Sexual partners* (n=229)	One sexual partner	17	160	0.51	0.21-1.22	0.124
	More than one partner	9	43			
*Polygamy* (n=232)	Polygamous	14	94	1.53	0.66 - 3.53	0.316
	Non-polygamous	11	113			
*Exposure to Surgery* (n=270)	Exposed	10	80	0.95	0.30 - 2.60	0.900
	Not Exposed	21	159			
*Blood Transfusion* (n=259)	Transfused	3	13	1.77	0.75–4.61	0.100
	Not Transfused	28	215			
*Contact with a Hepatitis B infected* (n=239)	Contact	4	19	1.86	0.68–3.87	0.142
	No contact	22	194			
*Sexual Partners* (n=40)	Multiple	1	3	2.67	0.44 - 3.23	0.527
	Single partner	4	32			
*History of jaundice* (n=234)	Positive history	5	27	1.69	1.21–2.14	0.041
	Negative history	20	182			

## Discussion

This is the first study documenting sero-prevalence of HBV among pregnant women in the newly formed Republic of South Sudan. The study documents that 11% of pregnant women were positive for HBV surface antigen (HBsAg). This prevalence is much higher than earlier documented by Elsheikh, in his study of Hepatitis B and hepatitis C prevalence in pregnant Sudanese women in 2007, when South Sudan was still under Sudan [10]. This statistic also confirms that HBV prevalence in South Sudan remains high, as was earlier reported by McCarthy MC et al in 1994 [19]. This re-affirms the conclusion that Sudan generated information should not be used in policy and programming for the newly formed Republic of South Sudan.

The prevalence of HBV estimated by this study compares very well with what was reported by Bayo P, et al in Northern Uganda [21]. The near exact estimates of HBV prevalence in Northern Uganda and South Sudan is attributable to similarity in lifestyles, similar post-conflict study settings, similar study methods (participant selection from one referral and public hospital) and similar laboratory methods (using ELISA kits with similar sensitivity levels). The similarity in prevalence estimates suggests that data/evidence generated in Northern Uganda is a better guide to policy and programming in South Sudan.

The prevalence of HBV estimated by this study also compares well with what was reported in other African countries [22–24]. While making comparisons between studies, we have cautiously considered a) Differences in methods used to detect antibodies (ELSIA versus DNA studies), b) socio-demographic variations between countries, c) laboratory testing algorithms and methods and d) post-conflict settings and influences on people behaviour.

For purposes of informing national policies on Hepatitis B vaccination, this study was conceptualized to provide evidence on introduction of Hepatitis B vaccines, either as a pentavalent combination with Diphtheria, Tetanus, Pertussis and Haemophilus Influenzae type B or a combination of pentavalent and monovalent Hepatitis B birth dose. The study confirms that there is a high-risk of vertical transmission of HBV infection to the unborn child in South Sudan to justify a monovalent birth dose introduction. Only 28.9% of pregnant women were immune (natural or vaccine induced) to Hepatitis B virus infections. Notably, 11% of children would be borne to mothers with acute/chronic Hepatitis B infection. Regrettably, this study did not screen HBsAg positive mothers for HBeAg that would inform determination of risks (probability) of vertical transmission. In the current health policy setting of South Sudan, pregnant women are not routinely screened for HBsAg, and the exposed newborns are not immunized at birth against HBV infection. These high prevalence rates of HBsAg among asymptomatic pregnant women in South Sudan suggests that the current vaccination policy is likely to leave out many infants born at high risk of vertical transmission and in turn at risk of becoming chronic hepatitis B carriers. A policy review on Hepatitis B vaccination is recommended with a view to introduce Hepatitis B birth-dose vaccination of infants [25, 26].

Although this study was not powered to investigate risk factors for HBV infections, we documented that only two out of the 10 factors we studied were significantly associated with positive HBsAg. The two factors were loss of a marital partner and history of Jaundice. We considered that loss of partner in the social-cultural context of South Sudan, exposes the women to multiple sexual partners. It’s important to note that sexuality is not a freely discussed topic in South Sudan.

In the meantime, the association of history of jaundice with HBsAg suggests that pregnant mothers can positively identify yellowing of eyes associated with HBV infections. Therefore history of jaundice could be an important variable for designing HBV birth-dose vaccination programs. In a resource constrained Public Health system of South Sudan, verbal screening of history of Jaundice could also be a simple tracer indicator for identifying mothers at high risk of vertical transmission of HBV. Such high-risk pregnant mothers could in turn be prioritized for birth dose vaccination using monovalent Hepatitis B vaccines.

Other risk factors considered important in Hepatitis B transmission were found insignificant in this study. The insignificant associations of HBsAG with parity, age, blood transfusion, circumcision/scarification, surgery, sexual partners and history of contact with HBV infected persons is similar to what was documented in Northern Uganda [21] and in Sudan [10, 19, 26]. These risk factors need further investigations in a Hepatitis B risk-factors study powered for this purpose and nationally representative samples before these conclusions are confirmed.

Like many other HBV sero-studies, our study had some limitations of: 1) Selection of study participants was in a referral/teaching hospital which could have biased the selection of pregnant mothers to those with pregnancy related complications. A similar study in lower levels of maternity service delivery points is highly recommended. 2) The study did not provide for high resolution abdominal ultrasound scans; and neither did we carry out serial liver enzyme tests to determine which mothers had active hepatitis B infections. Such additional tests would have added the ethical value of benefitting the infected mothers to obtain treatment for themselves. Understanding the limitations in the study settings and the existing maternal and child health services in South Sudan, we only limited the support to referring every mother that tested positive for HBsAg to a competent physician for a detailed consultation. 3) The study did not test for HBV DNA; and so there could have been HBsAg negative individuals with isolated anti-HBc and occult HBV infections that may not have been detected using the ELISA methods. We can only rest assured that pregnant women with very low HBV DNA levels would in turn have very low risk of transmitting HBV to their infants.

## Conclusion

These study findings suggest that only 30% of infants in Juba county are born to immune mothers (naturally or vaccine induced). The remaining 70% of babies would be at risk of infection, if a birth dose of Hepatitis B is not provided. We therefore recommended introduction of Hepatitis B Vaccine birth dose into routine infants’ vaccination series to eliminate this risk [27].

### What is known about this topic

- Hepatitis B virus infection is a major public health problem worldwide;
- Vertical transmission of HBV is the main cause of chronic HBV infection and is particularly a problem in endemic areas;
- Risk of vertical transmission of Hepatitis B can be reduced by Hepatitis B birth-dose policy.

### What this study adds

- This is the first ever study that documents the prevalence of Hepatitis B amongst pregnant women in the newly formed Republic of South Sudan;
- Documents that only 29% of infants in Juba county are born to immune mothers (27.1% naturally and 1.8% vaccine induced);
- Provides justification for introduction of the Hepatitis B birth-dose as a public health policy option that would contribute to reduction of risks for vertical transmission of Hepatitis B in South Sudan.
